# Evolution of the combined effect of different irrigation solutions and activation techniques on the removal of smear layer and dentin microhardness in oval-shaped root canal: An *in vitro* study

**DOI:** 10.17305/bjbms.2022.7440

**Published:** 2023-01-06

**Authors:** Lu Shi, Jie Wan, Yunfei Yang, Ying Yao, Ruiming Yang, Wen Xie

**Affiliations:** 1 Department of Endodontics, The First Affiliated Hospital of Zhengzhou University, Zhengzhou, Henan Province, People’s Republic of China

**Keywords:** Root canal therapy, photon-induced photoacoustic streaming, passive ultrasonic irrigation, smear layer, dentin microhardness

## Abstract

The aim of this *in vitro* study was to evaluate the effect of three final irrigants, namely QMix, MTAD, and EDTA, combined with three irrigation techniques, namely conventional needle irrigation (CNI), passive ultrasonic irrigation (PUI), and photon-induced photoacoustic streaming (PIPS), on smear layer removal, dentin mineral content, and microhardness in oval-shaped canals. One hundred and thirty decoronated premolars with single, oval root canals were equally divided into 1 blank control group and 12 treatment groups (*n* = 10) according to the final irrigation protocols. Roots in treatment groups were instrumented with ProTaper Gold to size F4 and subjected to final irrigation. Smear layer removal was assessed by using a four-level scoring system under an environmental scanning electron microscope. Energy dispersive X-ray spectroscopy was performed to measure the dentin mineral content. Dentin microhardness was measured by Knoop microhardness testing. Statistical analysis of the data was performed by using Kruskal–Wallis test, followed by Dunn’s *post hoc* test with Bonferroni correction. PUI- and PIPS-activated QMix and EDTA removed smear layer more effectively than MTAD groups (*p* < 0.05). Regarding the dentin mineral content and microhardness, QMix groups yielded the least calcium (Ca), phosphorus (P), and Ca/P ratio, followed by EDTA groups and MTAD groups (*p* < 0.05). QMix groups produced significantly lower dentin microhardness values and higher hardness reduction percentages than MTAD groups (*p* < 0.05). Within the limitations of the present study, it was concluded that QMix and EDTA were superior to MTAD in smear layer removal, especially when activated by PUI and PIPS, but these agents produced more pronounced effect on dentin mineral content and microhardness than MTAD.

## Introduction

The success of root canal treatment is fundamentally based upon effective infection control. However, apart from the microbiological challenge, the anatomical complexities can be further major obstacles in achieving these goals, such as accessory canals, ramifications, intercanal connections, fins, isthmuses, apical deltas, and recesses from C-shaped or oval/flattened canals [1, 2]. These irregular areas are difficult to be cleaned by mechanical preparation. Moreover, the debris and necrotic pulp tissues trapped in these hard-to-reach areas may block the entry of the irrigant flow, thus weakening the disinfection effect of chemomechanical preparation [2]. According to Jou et al., the oval-shaped canal was defined as having the maximal initial horizontal dimensions greater than the minimal initial dimension (up to two times more) at different levels of the canal [3]. Oval-shaped canals were present in 45% at the apical, 50% at the middle, and 56% at the coronal level of all teeth [4]. Due to the inconsistency between the irregular morphology and the round preparations provided by rotary instrumentation systems, 59.6%–79.9% of the dentinal walls remain untouched during the root canal preparation of oval-shaped canals [1, 5]. Bacteria and debris hidden in the unprepared areas can be the source of persistent infection and lead to the failure of root canal therapy. Thus, the problem of cleaning and disinfecting oval canals remains as one of the most significant clinical challenges as a result of the anatomical complexities. Moreover, endodontic smear layer is formed over the surface of dentinal wall during the root canal instrumentation, which is composed of dentin, remnants of pulp tissue, odontoblastic processes, and microorganisms [6]. It has been shown that this amorphous, irregular, and granular layer may delay the action of endodontic disinfectants and interfere with adhesion and penetration of sealers into dentinal tubules [6]. Therefore, irrigation is considered as a consequential clinical part of cleaning and shaping to facilitate the removal of necrotic tissue and dentin debris from mechanically prepared areas, as well as from the unprepared sites.

Sodium hypochlorite (NaOCl) is the most common irrigating solution used in endodontics due to its antimicrobial action and solvent capacity on organic tissues. Nonetheless, the use of NaOCl alone may leave the smear layer intact, as NaOCl has no effect on inorganic component of the smear layer. Final irrigation with 17% ethylenediaminetetraacetic acid (EDTA) is recommended to chelate and remove the mineralized portion of the smear layer [7]. More recently, compound solutions have been introduced as final irrigants. MTAD (Dentsply Tulsa Dental Specialties, Tulsa, OK, USA) contains 3% doxycycline, 4.25% citric acid, and 0.5% polysorbate 80 detergent (Tween 80), prepared by mixing the powder and the liquid before use [8]. In this formula, doxycycline is the main source of its antibacterial properties. Tween 80 helps to reduce surface tension and promote the penetration of MTAD into root canal irregularities and dentin tubules. Regarding the smear layer removal, citric acid serves as the demineralizing agent. Besides, doxycycline can act as a calcium chelator due to its low pH [9]. QMix^®^ 2 in 1 (QMix, Dentsply Tulsa Dental Specialties) is a premixed solution composed of EDTA, chlorhexidine (CHX), and a detergent (cetrimide). Nogo-živanović et al. [10] showed that QMix removed significantly more smear layer than 17% EDTA, but similarly to MTAD. On the contrary, MTAD showed the maximum removal of the smear layer in lower premolars, followed by ETAD and QMix [11]. According to the published data, no conclusive results could be drawn in a systematic review regarding the smear layer removal ability of the three chelating agents due to conflicting results [12].

To overcome the shortcomings of conventional needle irrigation (CNI) and improve the flushing effect, irrigation activation technology has been widely used in clinic. Passive ultrasonic irrigation (PUI) represents one of the most used systems to improve the endodontic irrigants activity. A systematic review demonstrated that ultrasonic activation was more effective than CNI in the removal of pulp tissue remnants and dentin debris based on both clinical and *in vitro* studies [13]. Photon-induced photoacoustic streaming (PIPS) technique utilizes Er:YAG laser system (2940 nm) equipped with a tapered radial and stripped tip [14]. Using 20 mJ per pulse, 15 Hz, and 50 µs pulse duration, a profound photoacoustic shock is created and streams irrigants three-dimensionally throughout the root canal system [14, 15]. Numerous *in vitro* experiments have been conducted to compare PIPS with other activation techniques in terms of smear layer removal, mostly with PUI [16]. Among these studies, 1%–6% NaOCl and 17% EDTA were the most commonly adopted irrigating solutions [16]. Investigations on the combined effect of different irrigation activation techniques and final irrigation with MTAD, QMix, and EDTA on removing of smear layer are scarce.

The occurrence of vertical root fracture (VRF) was more common in endodontically treated teeth (ETT) than in teeth with (non-)vital pulps. A retrospective study on 304 teeth with VRFs found that 295 (97%) teeth were ETT, while 7 (2.3%) were vital and 2 (0.7%) were non-vital but without caries [17]. It was reported that the prevalence of VRFs in ETT ranged from 4% to 32% [18]. The etiology of VRF is multi-factorial and can be broadly classified as predisposing risk factors and contributory risk factors [18]. Among these, changes in biomechanical properties of dentine were considered a predisposing risk factor. The use of intracanal disinfectants and medicaments may reduce the microhardness, elastic modulus, and fracture resistance of the dentin; moreover, prolonged exposure to these agents may further increase susceptibility to VRF [18]. According to Uzunoglu et al. [19], the mean fracture resistance of teeth treated with 17% EDTA for 10 min was about 2 times less than teeth treated with 5% EDTA for 10 min. Although the influences of root canal irrigants on dentin composition and microhardness have been extensively investigated, the results are inconsistent due to the heterogeneity of experimental design [20–23]. Furthermore, whether the effect of irrigants on these parameters varies according to the activation mode remains controversial [22, 23]. Such investigations are essential to strategize irrigation protocols with the better capacity for smear layer removal and less impact on dentin characteristics, such as microhardness, elastic modulus, fracture resistance, etc. Therefore, the aim of this study was to investigate the combined effect of irrigation activation techniques (PUI and PIPS) and final irrigation with MTAD, QMix, and EDTA on dentin in oval-shaped canals. The null hypothesis tested was that there would be no difference among the final irrigation protocols in terms of smear layer removal, dentin mineral content, and microhardness.

## Materials and methods

### Tooth selection and grouping

The sample size was calculated by using G Power Software with the effect size of 0.4 and *α*= 0.05 [24]. One hundred and thirty samples were required, 10 per group, to set the power to 80%.

Single-rooted and single-canaled mature maxillary and mandibular premolars with a curvature of less than 10∘ (measured according to the Schneider method [25]) extracted for orthodontic reasons were collected at the Department of Oral Surgery, the First Affiliated Hospital of Zhengzhou University (Zhengzhou, China), which belonged to 25 males and 27 females, with an age range between 18 and 25 years. Single, oval root canal morphology was confirmed by radiographs made in a buccolingual and mesiodistal direction. Teeth that had a buccolingual dimension two times greater than the mesiodistal dimension were considered oval canals [3]. The exclusion criteria were teeth with open apex, previous root canal treatment, calcification, and resorption. After calculus and soft tissue removal by curettes, the selected teeth were stored in 0.1% thymol solution at 4^∘^C for <2 months until further processing.

Dental crowns were sectioned with a high-speed diamond bur (SF-41, MANI, INC., Utsunomiya, Japan) under cooling with water spray to obtain 16 mm of root length from the anatomic apex. Ten of the decoronated teeth without further treatment were randomly selected by using a random number table and used as a blank control for energy-dispersive X-ray spectroscopy (EDX) and microhardness evaluation. By using the random number remainder grouping method, the remaining 120 teeth were randomly distributed into four experimental groups according to the final irrigant adopted: distilled water (DS) group, QMix group, EDTA group, and MTAD group. Subsequently, each group was further divided into three subgroups (*n* = 10) according to the irrigation activation technique employed: CNI, PUI, and PIPS. Groups with DS as the final irrigant were identified as the negative control.

### Root canal preparation

Working lengths of the 120 roots in the experimental groups were determined by subtracting 1 mm from the length at which the #10 K-file (Dentsply Maillefer, Ballaigues, Switzerland) first appeared at apical foramen. Then, the apical foramen of each canal was sealed with sticky wax to create a closed-end system.

Root canal preparation was initiated with glide path management by manipulating ProGlider instrument (tip size/taper: #16/.02) (Dentsply Sirona, York, PA, USA) at 300 rpm and a torque of 4.0 Ncm to full working length. Then, the root canals were shaped by using ProTaper Gold NiTi instruments to F4 (tip sizes/taper: #40/0.06) (Dentsply Sirona). The files were powered by an electric motor (X-Smart plus, Dentsply Maillefer) with the manufacturer’s recommendations. Throughout the period, 2 mL 5.25% NaOCl (except for the MTAD groups, where 2 mL 1.3% NaOCl was used) (all manufactured by Wuhan Longly Biotechnology Co., Ltd., Wuhan, China) was used as an intracanal irrigant solution by using a 30-G side-vented needle (Navitip, Ultradent, South Jordan, UT, USA) after each instrument. After the preparation, the canals were irrigated with 5 mL distilled water to rinse out NaOCl and dried with #40 paper points (Dentsply Maillefer).

### Final irrigation protocols

For each group, the total irrigant volume and total irrigant delivery time were standardized at 5 mL and 5 min, respectively.

For CNI (no-activation), the root canal was completely filled with 1 mL final irrigants, by using a 5 mL syringe with a 30-G side-vented needle (Navitip). The solution was remained in the canal for 5 min. In the last 20 s, the remaining 4 mL solution was expressed into the root canal by inserting the needle to 1 mm short of the working length and moving in an up-and-down motion with amplitude of 1–2 mm. No additional activation of irrigants was performed.

For PUI activation, after the placement of 1 mL final irrigants in the canal for 2 min, an endodontic irrigation tip IrriSafe^®^ #20/0.00 (Satelec^®^ , Acteon, Mérignac, France) was inserted to 1 mm short of the working length into the canal. The tip was powered by ultrasonic unit P5 Newtron^®^ XS (Satelec^®^ , Acteon) at power setting 5. In-and-out movements with amplitude of 2 mm were performed. The irrigant was activated for 30 s with a resting time of 30 s after activation. The applications were repeated three times. During the activation procedure, 4 mL solution was constantly replenished into the canal through the root canal opening.

For PIPS activation, 1 mL final irrigants were placed in the canal for 2 min. Then, an Er:YAG laser with a wavelength of 2940 nm (Fidelis AT, Fotona, Ljubljana, Slovenia) was used with a 14-mm long, 300-µm diameter quartz laser tip. The laser parameters were 0.3 w power, 15 Hz frequency, 20 mJ/pulse, and 50 µs pulse duration with the laser system water and air turned off. The tip was positioned at the canal entrance, and remained stationary during activation. The irrigant was activated for 30 s with a resting time of 30 s after activation. The applications were repeated three times. During the activation procedure, 4 mL solution was constantly replenished into the canal through the root canal opening.

After these procedures, the canals were immediately flushed with 5 mL distilled water and dried with #40 paper points.

Under the magnification with a dental operating microscope (Leica M525F20, Leica Microsystems GmbH, Wetzlar, Germany), two parallel longitudinal grooves on the buccal and lingual aspects of each root were made by using a high-speed diamond bur (SF-41, MANI) under the water-cooling without perforating the canal. A red-colored gutta-percha cone (tip size/taper: 25#/0.04, Dentsply Sirona) was placed inside the canal and the orifice was sealed with a small cotton plug. The gutta-percha cone was used as an indicator for the groove depth to avoid any intrusion of the bur into the canals hence avoiding any contamination due to debris produced by sectioning [26]. Then, the root was divided into two parts longitudinally along the grooves with a chisel (Shanghai Weirong Medical Equipment Co., Ltd., Shanghai, China). One randomly chosen half of each specimen was coded and subjected to environmental scanning electron microscopy (ESEM) and EDX examination. The other half of each specimen was subjected to microhardness evaluation. All the measurements were performed at three levels: coronal (11–13 mm from apex), middle (6–8 mm from apex), and apical (1–3 mm from apex) thirds. At each third, three points were randomly selected and measured, without any overlap between them. The representative values were obtained as the average of the results for the three measurements.

### Environmental scanning electron microscopy examination

The specimens were mounted on a stub and examined with an ESEM (Hitachi SU8010, High-Technologies Corp., Tokyo, Japan) at 1.0 kV. Photographs were taken at a magnification of 1000×. Two calibrated examiners blinded to the groups independently scored the images using a four-level scoring system: 1 = no smear layer with all tubules open; 2 = minimum quantity of smear layer with over 50% of the tubules open; 3 = moderate quantity of smear layer with less than 50% of the tubules open; and 4 = heavy smear layer with almost all dentin tubules obstructed [27]. When disagreement occurred during evaluation, the image was jointly reviewed and a consensus was obtained between examiners. Before the proper evaluation, training was conducted on a randomly selected sample of 20% of the ESEM images for calibration purposes. Inter-examiner reliability was assessed using Cohen’s kappa tests. A kappa score of 0.83 was achieved following the calibration exercise.

### Energy-dispersive X-ray spectroscopy

Subsequently, the elemental analysis under high vacuum at 1000× magnification was performed by using an energy dispersive X-ray system (EX-250, Horiba, Ltd., Kyoto, Japan) which is attached to the ESEM. The element content in weight % of calcium (Ca) and phosphorus (P) on the surface of dentin of each sample was measured; then the Ca/P ratio was calculated.

### Microhardness evaluation

The microhardness of the root dentin was determined in each specimen with an automatic turret digital display microhardness tester (HVS-1000, Shanghai Optical Instrument Factory, Shanghai, China). The indentations were made with a Knoop’s diamond indenter on each specimen at depths of 100 µm from the edge of the canal lumen using 100-g load and a 15-s dwell time. The hardness reduction percentages were calculated using the following formula: % = [(Initial microhardness - Final microhardness)/Initial microhardness]×100; where initial microhardness is the microhardness value of the blank control specimen (no-treatment) and final microhardness is the microhardness value of the test specimen (treatment) [23].

### Ethical statement

The study protocol was approved by the Ethics Committee of Scientific Research and Clinical Trial, the First Affiliated Hospital of Zhengzhou University (code: 2021-KY-1093).

### Statistical analysis

Statistical analysis of the data was done by using IBM SPSS 21.0 software (IBM SPSS Inc., Armonk, NY, USA). Data were analyzed by non-parametric Kruskal–Wallis test followed by Dunn’s *post hoc* test with Bonferroni correction since the normality distribution was refused by the Shapiro-Wilk test. The testing was performed at the 95% level of confidence (*p* < 0.05).

## Results

### Smear layer removal

**Figure 1.**
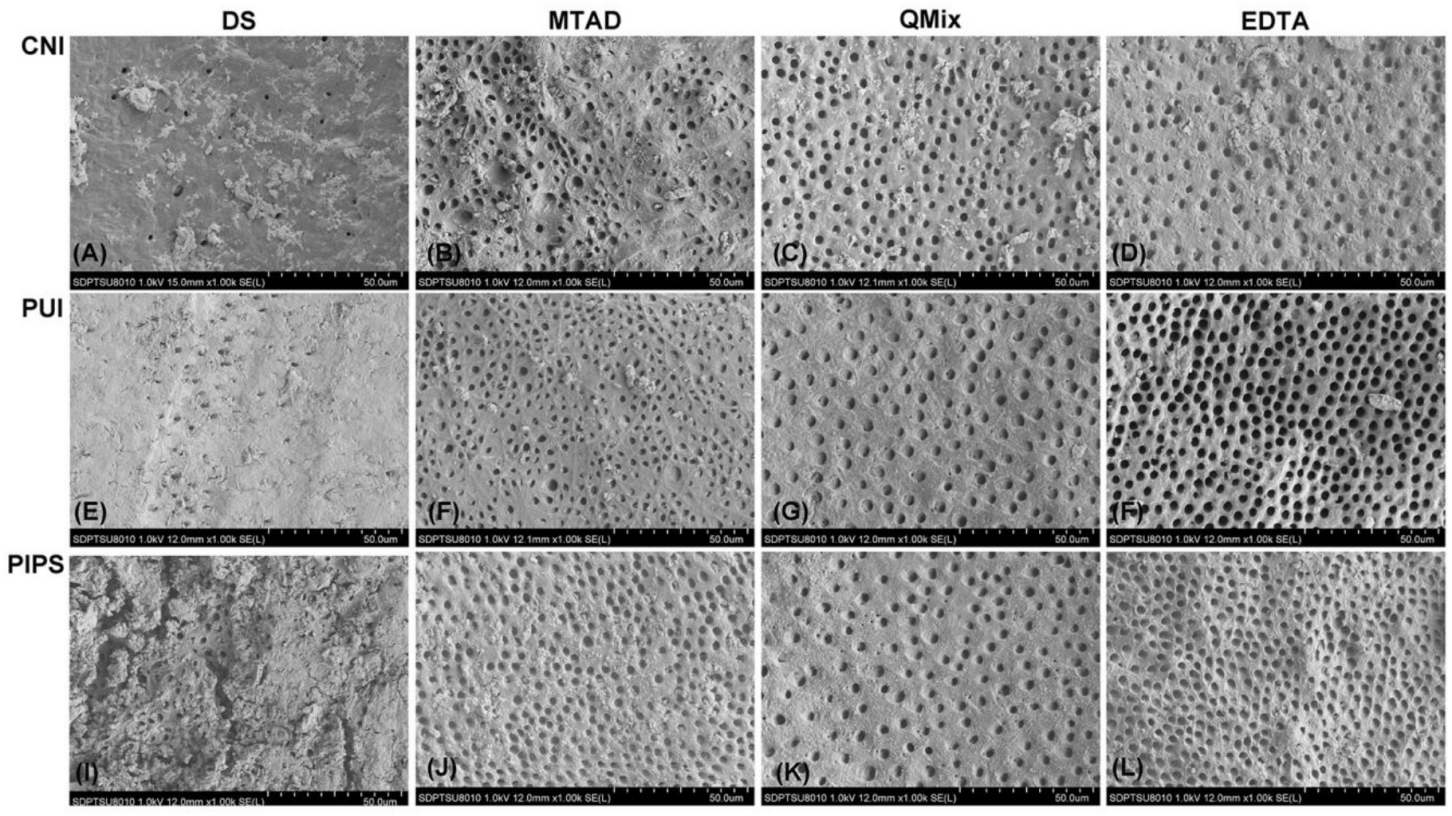
**Representative environmental scanning electron microscopy images of each group at the coronal third.** DS: distilled water, QMix: QMix 2 in 1, CNI: conventional needle irrigation, PUI: passive ultrasonic irrigation, PIPS: photon-induced photoacoustic streaming.

**Figure 2.**
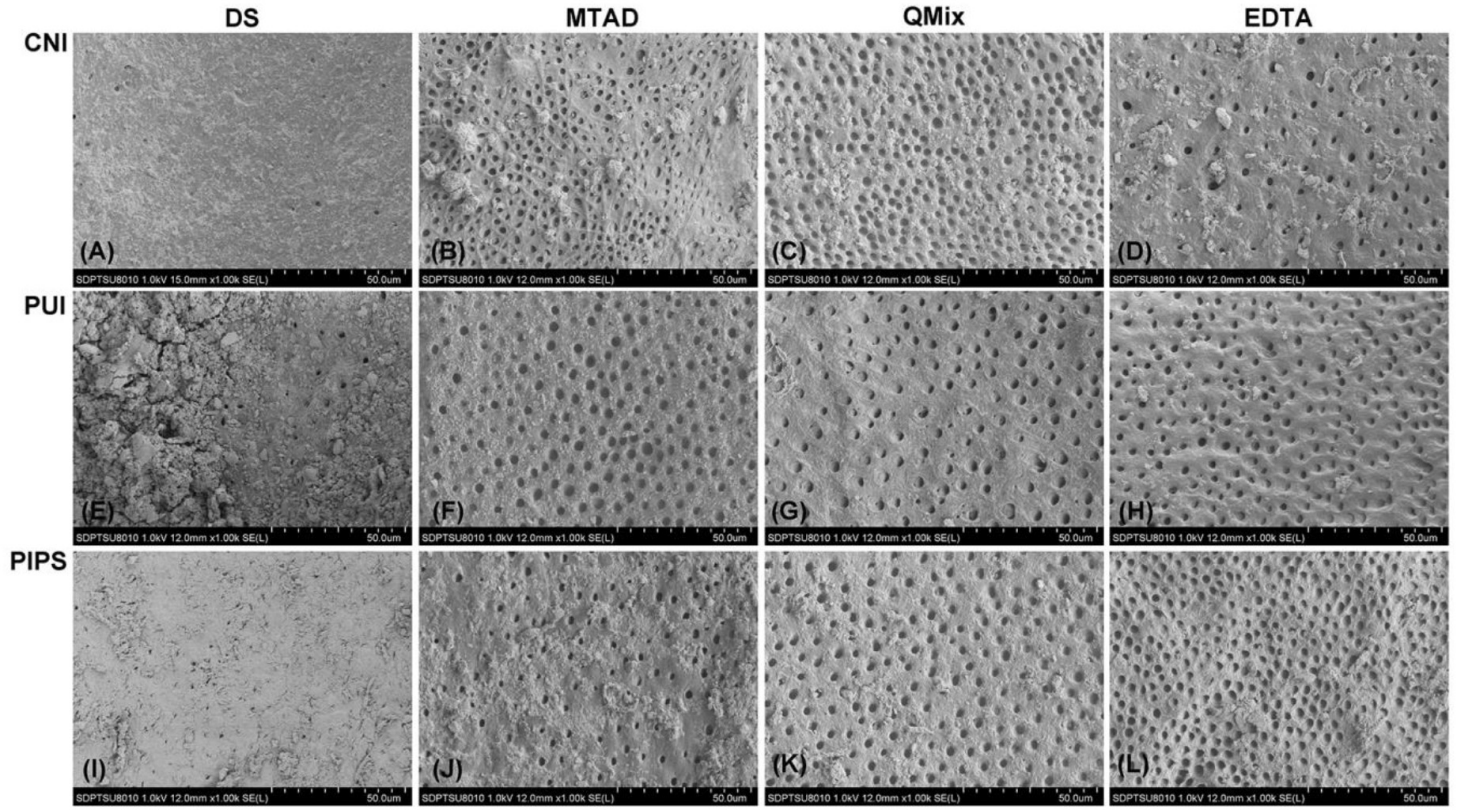
**Representative environmental scanning electron microscopy images of each group at the middle third.** DS: distilled water, QMix: QMix 2 in 1, CNI: conventional needle irrigation, PUI: passive ultrasonic irrigation, PIPS: photon-induced photoacoustic streaming.

**Figure 3.**
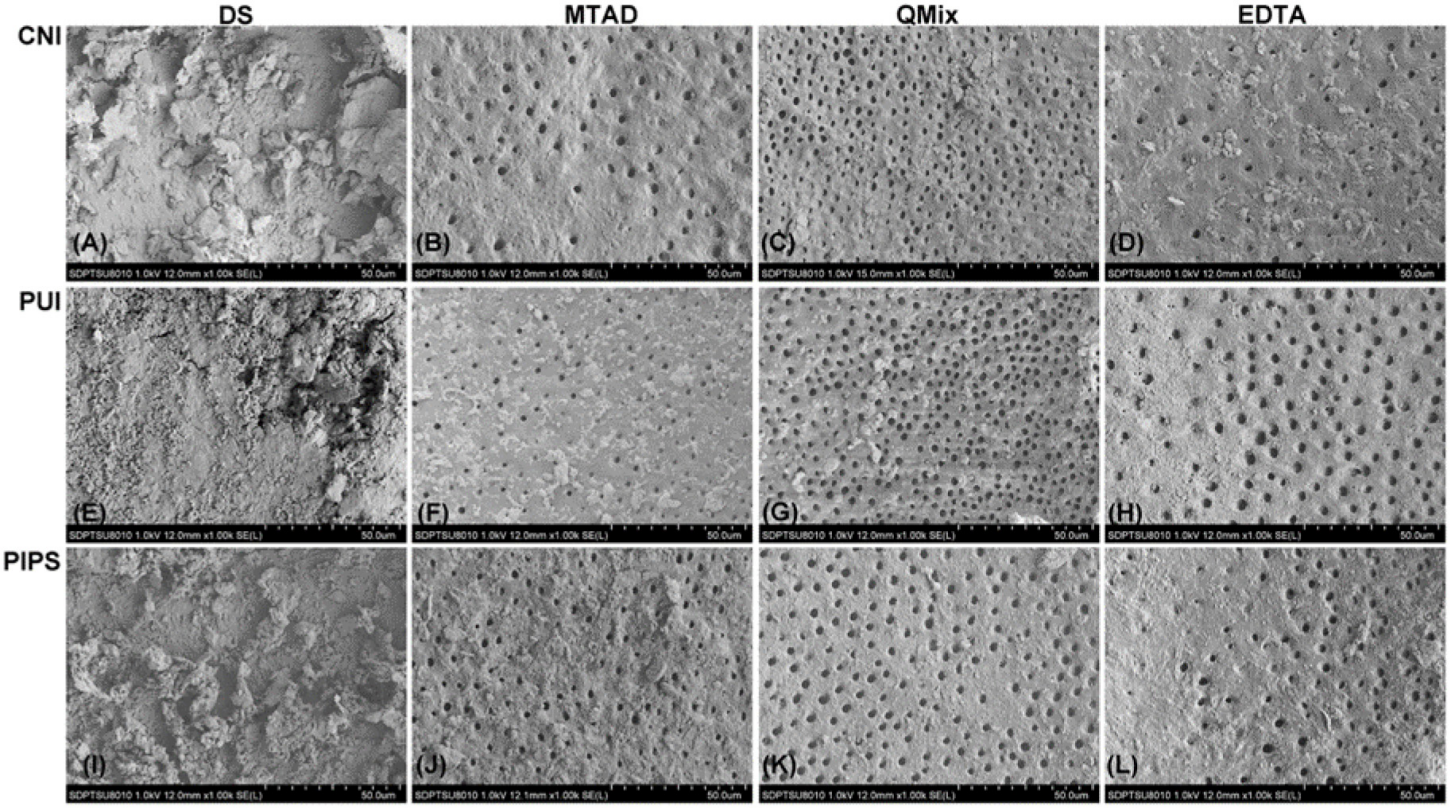
**Representative environmental scanning electron microscopy images of each group at the apical third.** DS: distilled water, QMix: QMix 2 in 1, CNI: conventional needle irrigation, PUI: passive ultrasonic irrigation, PIPS: photon-induced photoacoustic streaming.

**Figure 4.**
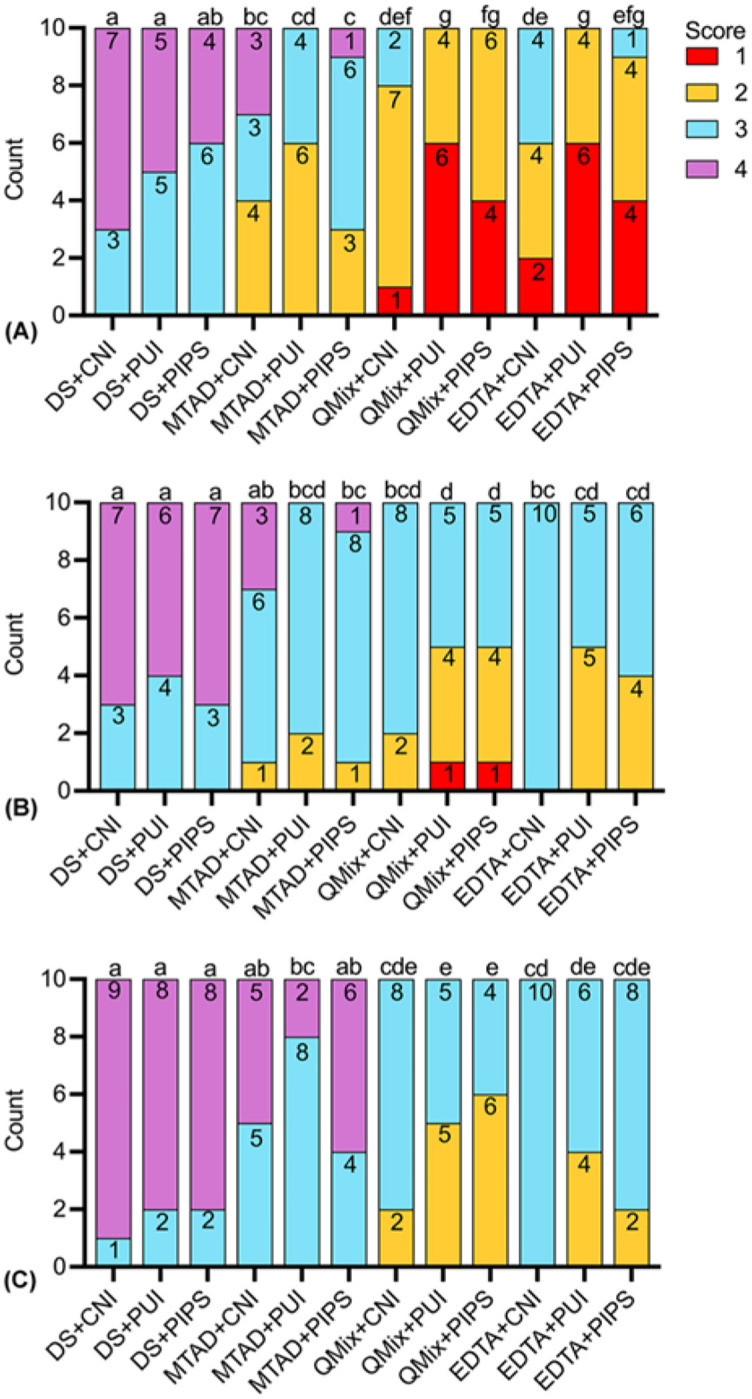
**The distribution of scores for the smear layer removal from oval-shaped root canal.** Different lowercase letters represent statistically significant difference between groups according to Kruskal–Wallis test followed by Dunn’s *post hoc* test with Bonferroni correction at α = 0.05. (A) Coronal third; (B) middle third; (C) apical third; DS: distilled water, QMix: QMix 2 in 1, CNI: conventional needle irrigation, PUI: passive ultrasonic irrigation, PIPS: photon-induced photoacoustic streaming.

The results of smear layer scores were presented in Table 1. Representative images for each group at coronal, middle, and apical third were illustrated in Figures 1–3, respectively. The distribution of smear layer scores was shown in Figure 4.

**Table 1:** Smear layer scores of root dentin samples at three thirds (upper quartiles, median, lower quartiles)

**Groups (n=10)**	**Coronal**	**Middle**	**Apical**
DS+CNI	^A^(3.25, 4, 4)^a^	^A^(3.25, 4, 4)^a^	^A^(4, 4, 4)^a^
DS+PUI	^A^(3, 3.5, 4)^a^	^A^(3, 4, 4)^a^	^A^(4, 4, 4)^a^
DS+PIPS	^A^(3, 3, 4)^ab^	^A^(3.25, 4, 4)^a^	^A^(4, 4, 4)^a^
MTAD+CNI	^A^(2, 3, 3.75)^bc^	^A^(3, 3, 3.75)^ab^	^A^(3, 3.5, 4)^ab^
MTAD+PUI	^B^(2, 2, 3)^cd^	^AB^(3, 3, 3)^bcd^	^A^(3, 3, 3)^bc^
MTAD+PIPS	^B^(2.25, 3, 3)^c^	^B^(3, 3, 3)^bc^	^A^(3, 4, 4)^ab^
QMix+CNI	^B^(2, 2, 2)^def^	^A^(3, 3, 3)^bcd^	^A^(3, 3, 3)^cde^
QMix+PUI	^B^(1, 1, 2)^g^	^A^(2, 2.5, 3)^d^	^A^(2, 2.5, 3)^e^
QMix+PIPS	^B^(1, 2, 2)^fg^	^A^(2, 2.5, 3)^d^	^A^(2, 2, 3)^e^
EDTA+CNI	^B^(2, 2, 3)^de^	^A^(3, 3, 3)^bc^	^A^(3, 3, 3)^cd^
EDTA+PUI	^B^(1, 1, 2)^g^	^A^(2, 2.5, 3)^cd^	^A^(2, 3, 3)^de^
EDTA+PIPS	^B^(1, 2, 2)^efg^	^A^(2, 3, 3)^cd^	^A^(3, 3, 3)^cde^

Different lowercase letters represent statistically significant difference among groups (column) and different capital letters represent statistically significant difference among thirds (row) according to Kruskal–Wallis test followed by Dunn’s *post hoc* test with Bonferroni correction at α = 0.05. DS: distilled water, QMix: QMix 2 in 1, CNI: conventional needle irrigation, PUI: passive ultrasonic irrigation, PIPS: photon-induced photoacoustic streaming.

Regarding the scores of smear layer removal among thirds in each group, coronal scores were significantly lower than middle and apical scores (*p* < 0.05), except for the negative subgroup (DS as the final irrigant) and the MTAD+CNI group, where no significant differences were found.

All the negative groups had a significantly higher score than all the QMix and EDTA subgroups in all thirds (*p* < 0.05). Activated MTAD subgroups produced a lower smear layer scores than all the negative subgroups in coronal and middle thirds (*p* < 0.05).

In the coronal third, PUI- and PIPS-activated QMix and EDTA removed significantly more smear layer compared with all the MTAD subgroups (*p* < 0.05). PUI-activated QMix and EDTA performed better than nonactivated QMix and EDTA (*p* < 0.05). In the middle third, PUI- and PIPS-activated QMix produced the lowest scores with significant difference compared with MTAD+CNI group, MTAD+PIPS group, and EDTA+CNI group (*p* < 0.05). In the apical third, PUI- and PIPS-activated QMix and EDTA performed similarly (*p* > 0.05) and they removed smear layer more effectively than all the MTAD subgroups (*p* < 0.05).

### Elemental analysis

**Figure 5.**
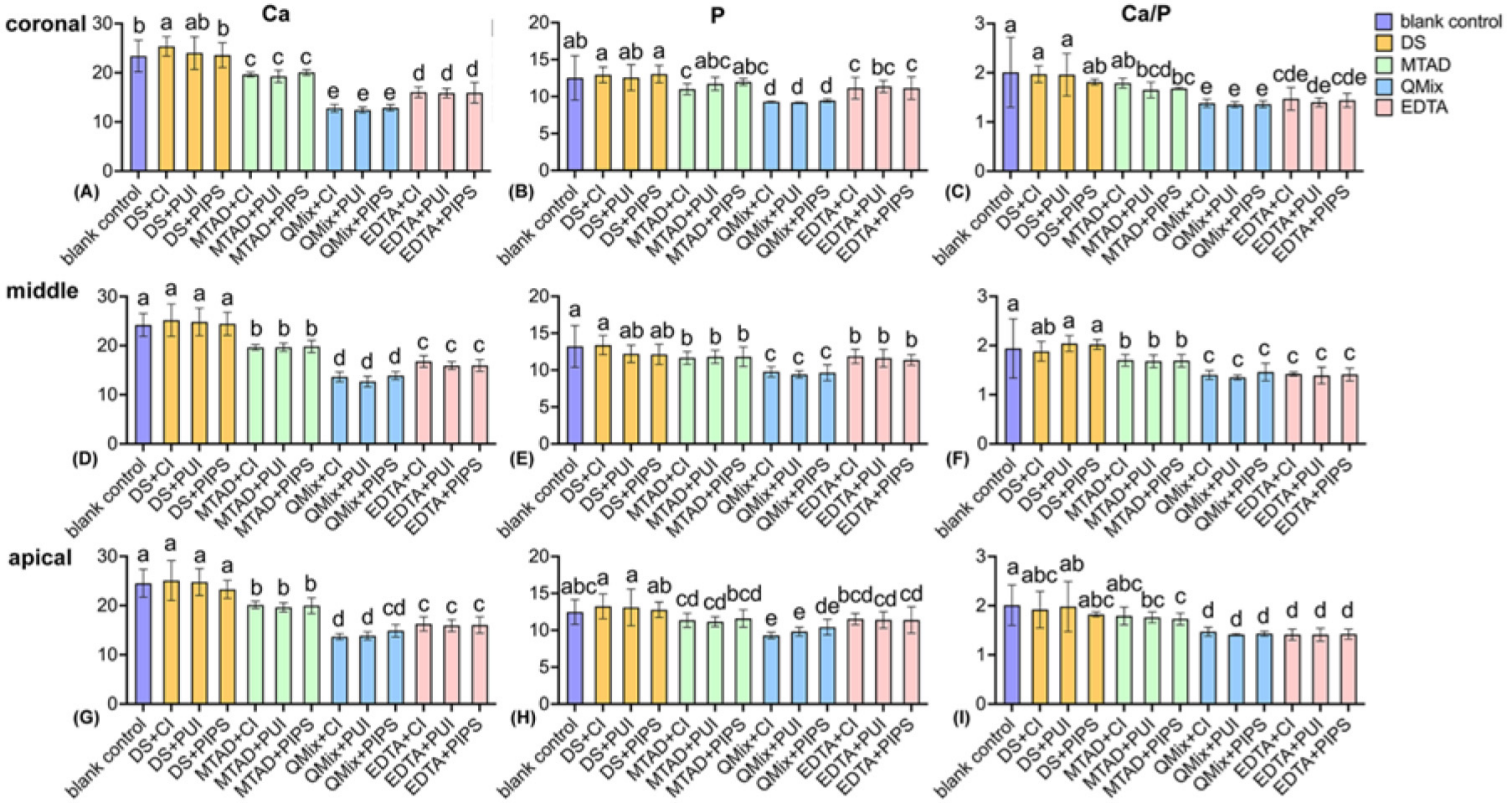
**The calcium (Ca), phosphorus (P) contents (weight %), and Ca/P (weight ratio) of root dentin samples at three thirds.** Different lowercase letters represent statistically significant difference between groups according to Kruskal–Wallis test followed by Dunn’s *post hoc* test with Bonferroni correction at α = 0.05. (A-C): Coronal third; (D-F): middle third; (G-I): apical third; DS: distilled water, QMix: QMix 2 in 1, CNI: conventional needle irrigation, PUI: passive ultrasonic irrigation, PIPS: photon-induced photoacoustic streaming.

The results of Ca (weight %) and P (weight %) contents and Ca/P ratio (weight ratio) of root dentin samples were shown in Figure 5.

In all thirds, the level of Ca content was in the following order: QMix groups < EDTA groups < MTAD groups < blank control and negative groups (*p* < 0.05), except for the QMix+PIPS group, which was similar to the EDTA groups in the apical third (*p* > 0.05). Intra-group comparisons showed that Ca content in the DS+PIPS group was slightly lower than that in the DS+CNI group in the coronal third (*p* < 0.05).

Compared with the negative groups and blank control, the level of P content in QMix, EDTA, and MTAD groups decreased in all thirds. QMix groups produced the significantly lowest level of P content in all thirds (*p* < 0.05), except for QMix+PIPS in the apical third, which was similar to the EDTA groups (*p* > 0.05). No statistically significant difference was found between MTAD groups and EDTA groups in all thirds (*p* > 0.05), except for the EDTA+PIPS group in the middle third, which produced a higher P content than MTAD groups, EDTA+CNI and EDTA+PUI groups in the middle third (*p* < 0.05).

Regarding the Ca/P ratio in all thirds, QMix groups and EDTA groups produced relatively lower values than MTAD groups (*p* < 0.05). No significant difference was found between QMix groups and EDTA groups (*p* > 0.05). In all thirds, there was no significant difference in intra-group comparisons (*p* > 0.05).

### Microhardness

**Figure 6.**
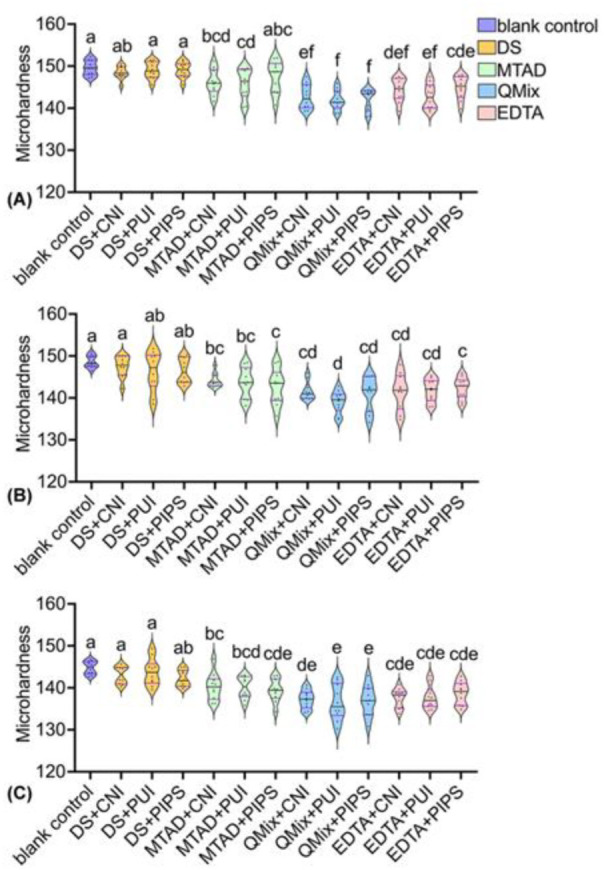
**Violin plots illustrating the microhardness values of each group.** Different lowercase letters represent statistically significant difference between groups according to Kruskal–Wallis test followed by Dunn’s *post hoc* test with Bonferroni correction at α = 0.05. (A) Coronal third; (B) middle third; (C) apical third; DS: distilled water, QMix: QMix 2 in 1, CNI: conventional needle irrigation, PUI: passive ultrasonic irrigation, PIPS: photon-induced photoacoustic streaming.

Violin plots illustrated the microhardness values among the different groups at three measuring levels (Figure 6). Compared with the blank control group and negative groups, the microhardness values in QMix groups and EDTA groups significantly reduced in all thirds (*p* < 0.05). The same was true for MTAD+PUI in coronal third, as well as for MTAD+PIPS in middle and apical third (*p* < 0.05). No significant difference was found in the intra-group comparisons in all thirds (*p* > 0.05).

In the coronal third, the microhardness values in QMix groups and EDTA+PUI group were significantly lower than that in MTAD+PUI group (*p* < 0.05). In the middle third, QMix+PUI group produced a significantly lower value than MTAD groups and EDTA+PIPS group (*p* < 0.05). In the apical third, there was no significant difference between MTAD groups and EDTA groups, nor between EDTA groups and QMix groups (*p* > 0.05). QMix+PUI group and QMix+PIPS group showed significantly lower values than MTAD+CNI group and MTAD+PUI group in the apical third (*p* < 0.05).

**Figure 7.**
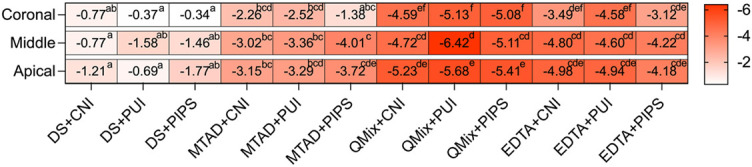
**Heatmap depicting the microhardness reduction percentages of each group at three thirds.** Different lowercase letters represent statistically significant difference between groups according to Kruskal–Wallis test followed by Dunn’s *post hoc* test with Bonferroni correction at α = 0.05. DS: distilled water, QMix: QMix 2 in 1, CNI: conventional needle irrigation, PUI: passive ultrasonic irrigation, PIPS: photon-induced photoacoustic streaming.

Heatmap depicted the microhardness reduction percentages of each group in all thirds (Figure 7). QMix+PUI group and QMix+PIPS group produced higher reduction percentages than other groups at each third with/without statistically significant difference (*p* < 0.05).

## Discussion

Previous studies have tested the ability of smear layer removal and effect on dentin microhardness of irrigants, including MTAD, QMix, and EDTA. However, because of the considerable heterogeneity in the methodologies, including types of root canal system and model, types of activation technique, irrigation times, irrigation solutions and their concentration, the outcomes of *in vitro* studies were conflict. Stimulating the *in vivo* conditions and standardization of the specimens and experimental protocols are fundamental requirements for studies on irrigants and irrigation systems [28]. Reassembling a split tooth embedded in a silicone mold was used in a previous study [23]. This open system model is not in line with the clinical situation. It has been underlined that during irrigation, the root canal behaves mostly like a closed-ended system. Closed apical foramen result in significantly more complex flow patterns and add considerable barriers to irrigant penetration compared to open systems [29]. The apical foramen was sealed with wax in the present study to mimic the clinical condition. Irrigant concentration, volume, and time are the key factors to determine the chemical effect of irrigation, whereas the mechanical effect is mainly influenced by the flow rate and the intensity of agitation [28]. Regarding the concentration of initial NaOCl, 5% NaOCl increased the smear layer removal efficiency of QMix as compared to 2.5% NaOCl concentration, while no significant difference was observed for EDTA solution [30]. According to Boutsioukis and van der Sluis [29], the effectiveness of MTAD for complete removal of the smear layer was enhanced when low concentrations of NaOCl (1.3%) were used as an intracanal irrigant before the use of MTAD as a final rinse. Meanwhile, this regimen does not seem to significantly change the structure of the dentinal tubules [31]. Consequently, 5.25% NaOCl was used between each file during the root canal preparation for EDTA groups and QMix groups, while 1.3% NaOCl for MTAD groups. The flow rate during CNI affects significantly the flow pattern within the root canal [32]. Unfortunately, this parameter was overlooked in most studies, and low flow rate was adopted [10, 22, 28]. Hardly any irrigant refreshment could be achieved apically to the needle when irrigating at a very low flow rate (0.02 mL/s), but a flow rate at 0.15–0.2 mL/s can provide refreshment up to 1 mm apically to the needle [32, 33]. On the other hand, the flow rate as high as 0.53–0.79 mL/s cannot be regarded as average clinical conditions, although it can provide refreshment up to 1.5 mm apically to the needle [32]. Moreover, the flow rate affects the velocity gradient near the root canal wall as well as the wall shear stress, which is responsible for the mechanical cleaning effect [33]. Therefore, a flow rate of 0.2 mL/s (4 mL in 20 s) combined with placing the irrigation needle within 1 mm from working length was adopted in the present study to achieve acceptable irrigant exchange [32]. Besides, other critical parameters, such as working length, apical size and taper, irrigant volume, and time, were also standardized for each group to increase the internal validity of the study [28]. ESEM was employed in the present study to assess the smear layer removal. Compared to SEM, ESEM is a non-destructive means and does not require any prior preparation of the specimens, such as dehydration and sputter coating, thus avoiding the possible damage to the sample [34]. However, the assessment is still two-dimensional and based on subjective scoring systems [28]. In the present study, a semi-quantitative scoring system was used and the observer calibration was conducted on 20% of the ESEM images to reduce the personal error.

The removal of smear layer in each group was significantly more effective in the coronal third than in the middle and apical third. Although no significant difference was found in MTAD+CNI group among thirds, relatively higher percentages of samples received score 4 in the apical third, indicating that heavier smear layer remained in this area. This finding is consistent with the previous studies showing that smear layer removal from the apical region remains a difficult problem to be solved [35, 36]. Irrigant exchange in the various parts of the root canal system is a crucial requirement for an adequate chemical and mechanical effect [29]. The reduced canal diameter in this region affects the dynamics of irrigant flow and subsequently the disinfecting and dissolution effects of irrigation [37]. Nevertheless, PUI- and PIPS-activated QMix and EDTA enhanced the removal of smear layer from apical area with more samples scored 2 compared with other groups.

The QMix contains cetrimide, a detergent that decreases surface tension and increases wettability and penetrability. It was suggested that low surface tension may facilitate the contact of irrigant solution with the dentinal walls, enhancing QMix effectiveness in smear layer removal [10]. However, inter-group comparisons demonstrated that QMix removed smear layer equally well as EDTA. Consistent with our results, the findings of Matos et al. also showed that there was no significant difference between EDTA and QMix regarding smear layer removal [38]. On the other hand, some studies found that QMix removed the smear layer more effectively than 17% EDTA [12]. In the present study, the cleaning efficiency of QMix and EDTA was similar or superior to that of MTAD groups, especially in the coronal and apical third. These results were in accordance with previous studies [9]. In turn, some studies found that MTAD showed statistically significant better cleansing effect than EDTA and QMix [39, 40]. It is to be noted that in these studies, higher concentration (3% and 5.25%) of initial NaOCl were adopted in all experimental groups [39, 40]. In the present study, the initial NaOCl concentration was 5.25% in the EDTA and QMix groups, while it was 1.3% in the MTAD group. Collagen degradation kinetics were more rapid and severe when 5.25% NaOCl was used as the initial irrigant compared to 1.3% NaOCl, and the apatite/collagen ratio increased, indicating more apatite than intact collagen within the dentin subsurface, thus exposing higher inorganic content to the external surface of the dentin [41]. Whether the difference in concentration of initial NaOCl adopted in QMix, EDTA, and MTAD groups could be a reasonable explanation for the inconsistent results deserves further research.

With the smear layer removal, irrigating solutions simultaneously cause alterations in the mineral content of dentin, which may decrease its microhardness. Therefore, mineral content and dentin microhardness were assessed subsequently in the present study. Published studies found that irrigants that remove more of the smear layer showed more changes in the microhardness of dentin [11]. In the present study, QMix and EDTA groups exhibited improved effective removal of smear layer; consequently, these groups also produced more alteration in the microhardness of dentin than MTAD groups. The reason may be attributed to the fact that with the removal of the smear layer, which may act as a physical barrier, the flushing fluid could contact the exposed dentin extensively, resulting in a more obvious decrease in microhardness. Panighi and G’Sell [40] found the microhardness of dentin increased linearly with the calcium concentration [42]. Taneja et al. [21] assessed the effect of chelating agents on the calcium loss and its subsequent effect on the dentin microhardness and found that a reduction in the microhardness of root dentin was observed with an increase in calcium loss from root dentin. This was further supported by the present study, showing that QMix and EDTA groups produced noticeable effects on the mineral content of root dentin compared with MTAD groups; accordingly, lower mean dentin microhardness and higher hardness reduction percentages were obtained in QMix and EDTA groups compared with MTAD groups with/without statistical significance.

Due to the different concentration and duration of initial NaOCl, plus the different final irrigation protocols used in the previous studies, the demineralization effect of the three chelating solutions adopted in the present study has not yet reached a consistent conclusion. Here, the inter-group comparison found that Ca decreased the most with QMix, followed by EDTA and MTAD. P reduced more with QMix with no difference between EDTA and MTAD. QMix groups and EDTA groups produced relatively lower Ca/P values than MTAD groups with no significant difference between QMix and EDTA groups. Similarly, Ballal’s research showed that Ca and P reduced more with QMix than EDTA [43]. On contrary, there was no significant difference between QMix and EDTA regarding calcium loss according to Taneja et al. [21]. Nogo-živanović et al. [10] found that MTAD yielded the most pronounced effect on the mineral content of root dentin compared with QMix and EDTA; however, these differences did not reach significance. In addition to the composition and duration of the chelating solutions, which could affect the demineralization effect, the concentration and duration of initial NaOCl are also important factors. Published studies investigated the effect of irrigation sequences involving NaOCl and EDTA on dentin composition and found that the sequences involving NaOCl, such as NaOCl/EDTA, EDTA/NaOCl, and NaOCl/EDTA/NaOCl, significantly decrease the Ca and P content of the dentin when compared to irrigation with EDTA alone [44]. Considering the possible internal relationship among the smear layer removal, microhardness and element composition discussed above, as well as the impact of NaOCl on these characteristics, future studies should include NaOCl treatment as a variable to explore the optimal combination of initial NaOCl and final irrigant to achieve effective cleaning of the smear layer with minimal changes in the dentin.

With regard to the influence of irrigation activation technique on the demineralization effect of the three chelating final irrigants, no significant difference could be drawn from the comparisons of intra-group findings, except for the level of Ca content in DS groups in the coronal third and P content in EDTA groups in the middle third. In consistent with our results, Akbulut and Terlemez [23] found that PIPS activation did not cause an additional decrease in dentin microhardness, indicating that the alterations in dentin mineral content and dentin microhardness were mainly affected by the irrigation solution, not by the activation technique. Differently, Quteifani et al. [22] found that Er:Yag laser-activation (2 w power, 15 Hz frequency) of irrigants resulted in significantly less reduction of micro-hardness when compared to no-laser-activation. This discrepancy may be related to the different laser parameters, which results in different degrees of changes in the chemical components [22]. The exceptions noted in PIPS-activated DS and EDTA may be contributed to the fact that laser applications in root canals can cause some morphological and chemical changes in root canal dentin [45]. The severity of these changes depends on the type of laser energy as well as the density and absorption characteristics of the tissue [45]. Confirming our results, Akbulut and Terlemez [23] found no alteration in dentin mineral content between the non-activated and PIPS-activated groups, except for the NaOCl+PIPS group, in which the PIPS-activated NaOCl significantly increased the P level [23]. The authors explained that PIPS could induce NaOCl to react with hydroxyapatite molecules or accelerate this chemical reaction, resulting in a higher P content [23]. Thus, the combined effect of PIPS and irrigants on changes in dentin composition warrants further study.

Further, the main deficiency of the present study is the *in vitro* experiment, which cannot fully simulate the complex oral environment. In the future, further *in vivo* research is needed to explore the clinical significance of the effect of these irrigation protocols on the ultrastructure of dentin. In addition, for oval-shaped canals, it is crucial to clean the recesses and un-prepared areas with irrigants. Thus, the penetration of the irrigants in the root canal system including the dentinal tubules and its impact on clinical treatment outcome should be investigated in future studies [46].

Taken together, within the parameters of this study, QMix and EDTA were more efficient than MTAD in smear layer removal, especially when activated by PUI or by PIPS. Meanwhile, QMix and EDTA produced more significant effects on dentin composition and microhardness than MTAD. The current results indicated that for the irrigation protocols investigated in this study, it is difficult to strike a balance between effective removal of the smear layer and minimal adverse effects on dentin.

## Acknowledgments

This work was supported by the joint construction project of the medical science and technology research plan of Henan Province (Grant numbers. LHGJ20190197).

**Conflicts of interest:** The authors declare no conflicts of interest.

**Funding:** This work was supported by the joint construction project of the medical science and technology research plan of Henan Province (Grant numbers. LHGJ20190197).
